# Synthesis, anti-inflammatory, cytotoxic, and COX-1/2 inhibitory activities of cyclic imides bearing 3-benzenesulfonamide, oxime, and β-phenylalanine scaffolds: a molecular docking study

**DOI:** 10.1080/14756366.2020.1722120

**Published:** 2020-02-03

**Authors:** Alaa A.-M. Abdel-Aziz, Adel S. El-Azab, Nawaf A. AlSaif, Mohammed M. Alanazi, Manal A. El-Gendy, Ahmad J. Obaidullah, Hamad M. Alkahtani, Abdulrahman A. Almehizia, Ibrahim A. Al-Suwaidan

**Affiliations:** Department of Pharmaceutical Chemistry, College of Pharmacy, King Saud University, Riyadh, Saudi Arabia

**Keywords:** Cyclic imide, anti-inflammatory activity, cytotoxic activity, COX-1/2 inhibition, molecular docking

## Abstract

Cyclic imides containing 3-benzenesulfonamide, oxime, and β-phenylalanine derivatives were synthesised and evaluated to elucidate their *in vivo* anti-inflammatory and ulcerogenic activity and *in vitro* cytotoxic effects. Most active anti-inflammatory agents were subjected to *in vitro* COX-1/2 inhibition assay. 3-Benzenesulfonamides (**2–4**, and **9**), oximes (**11–13**), and β-phenylalanine derivative (**18**) showed potential anti-inflammatory activities with 71.2–82.9% oedema inhibition relative to celecoxib and diclofenac (85.6 and 83.4%, respectively). Most active cyclic imides **4**, **9**, **12**, **13**, and **18** possessed ED_50_ of 35.4–45.3 mg kg^−1^ relative to that of celecoxib (34.1 mg kg^−1^). For the cytotoxic evaluation, the selected derivatives **2–6** and **8** exhibited weak positive cytotoxic effects (PCE = 2/59–5/59) at 10 μM compared to the standard drug, imatinib (PCE = 20/59). Cyclic imides bearing 3-benzenesulfonamide (**2–5**, and **9**), acetophenone oxime (**11–14**, **18**, and **19**) exhibited high selectivity against COX-2 with SI > 55.6–333.3 relative to that for celecoxib [SI > 387.6]. β-Phenylalanine derivatives **21–24** and **28** were non-selective towards COX-1/2 isozymes as indicated by their SI of 0.46–0.68.

## 1. Introduction

**Figure 1.**
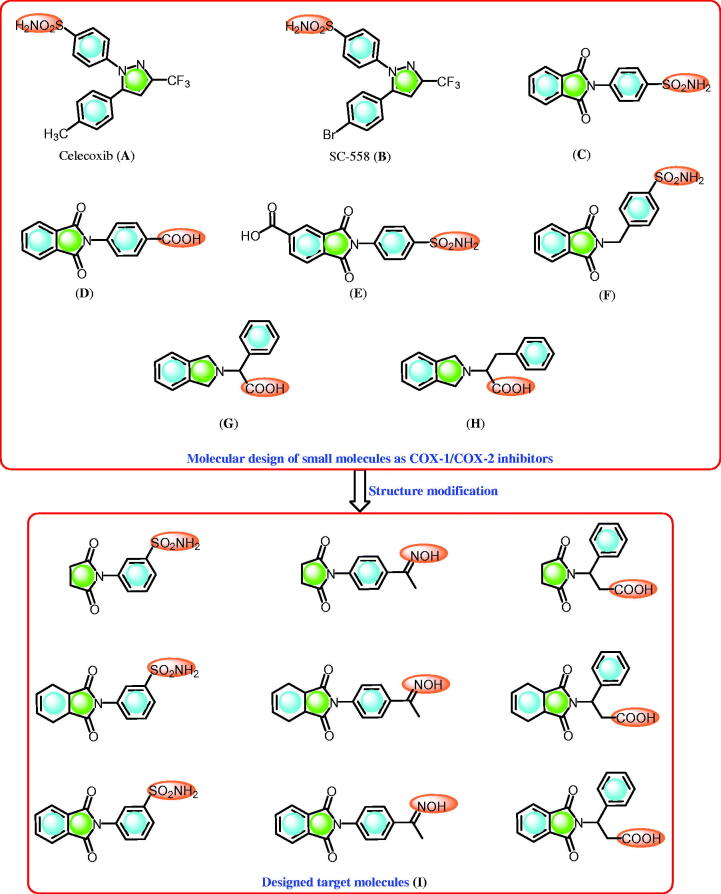
Some reported selective COX-2 inhibitors (A–H) and the designed compounds (I).

Nonsteroidal anti-inflammatory drugs (NSAIDs) are the drugs of choice for the treatment of inflammation and pain^1^^,^^2^. NSAIDs are cyclooxygenase inhibitors and they include the COX-1 and COX-2 enzymes^1–3^. The cyclooxygenase isozymes are responsible for the conversion of arachidonic acid to eicosanoids (prostaglandins)^1–3^. In this process, the inhibition of the COX-2 isozyme elucidates the anti-inflammatory effects of NSAIDs^1–3^ while the inhibition of the COX-1 isozyme is responsible for the major side effects displayed by NSAIDs, such as gastrointestinal implication^1^^,^^2^. Alternatively, selective COX-2 inhibitors, including compounds that contain the pyrazole ring system, such as celecoxib (A) and SC-558 (B)^4^ (Figure 1), display anti-inflammatory activity with improved gastric profile protection compared to NSAIDs^4^. Moreover, celecoxib, a COX-2 inhibitor (Figure 1(A)) that displays an anti-inflammatory effect, has been investigated as an antitumor agent^5^.

Compounds containing the sulphonamide moiety are interesting derivatives that possess versatile and potential biological activities, such as carbonic anhydrase inhibitors^6–9^, COX-1/2 inhibition (Figure 1(C–F))^6^^a,^^10^^,^^11^, and anti-inflammatory (Figure 1(C–F))^6^^a,^^10^^,^^11^ and antitumor activities^6^^a,^^12^. Recently, we reported the synthesis of phthalimide derivatives with potential anti-inflammatory^6^^a,^^10^^,^^11^, antitumor^6^^a,^^13^, hypoglycaemic, and antihyperlipidemic properties^14^. In addition, some of these compounds were identified to display potent COX-2 and carbonic anhydrase inhibitory activities^6–11^. Recently, phthalimide analogues such isoindolines bearing α-amino acids are reported as promising COX-1/COX-2 inhibitors (Figure 1(G,H))^15^.

Here, we report the design and synthesis of novel cyclic imides containing 3-benzenesulfonamide, acetophenone oxime, and β-phenylalanine scaffolds. The designed cyclic imides (Figure 1(I)) were subjected to various analyses to: (i) investigate their *in vivo* anti-inflammatory and ulcerogenic activities, and their *in vitro* COX-1/2 inhibitory effects; (ii) study their *in vitro* cytotoxicity activity; (iii) explore their structure-activity relationships (SAR) based on their *in vivo* anti-inflammatory effects and *in vitro* COX-1/2 inhibitory activity with molecules containing substituted cyclic imides; (iv) compare the biological effects of 3-benzenesulfonamide to those of acetophenone oxime and β-phenylalanine based on *in vivo* anti-inflammatory and COX-1/2 inhibition; and (v) conduct a molecular docking study of the target derivatives to investigate their binding with the COX-2 isozyme.

## 2. Results and discussion

### 2.1. Chemistry

**Scheme 1.**
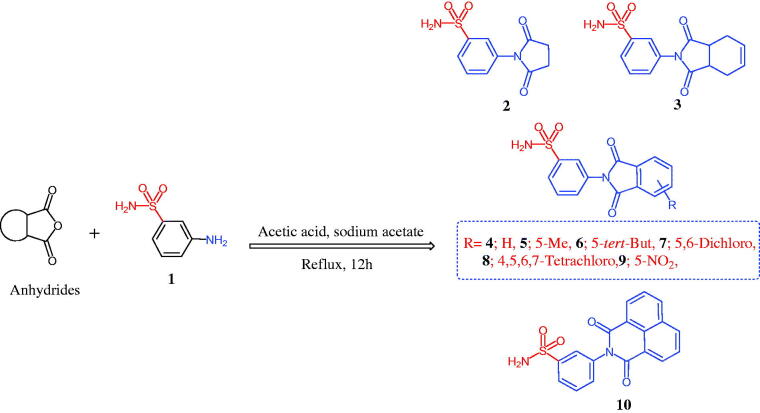
Synthesis of 1,3-isoindolinediones incorporating 3-benzenesulfonamide **2–10**.

**Scheme 2.**
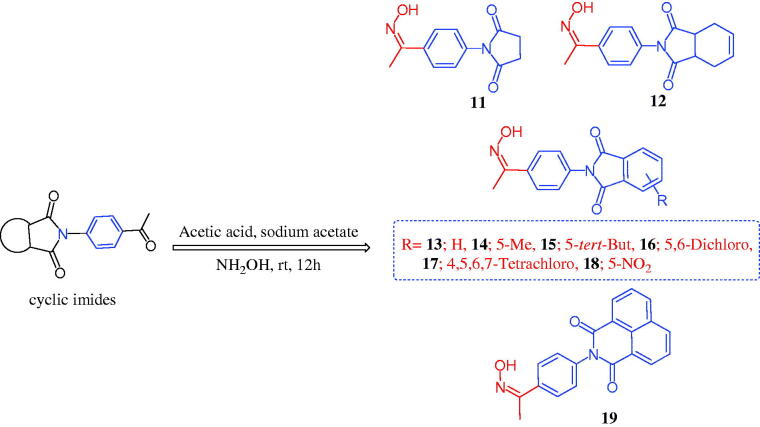
Synthesis of 1,3-isoindolinediones incorporating oxime **11–19**.

**Scheme 3.**
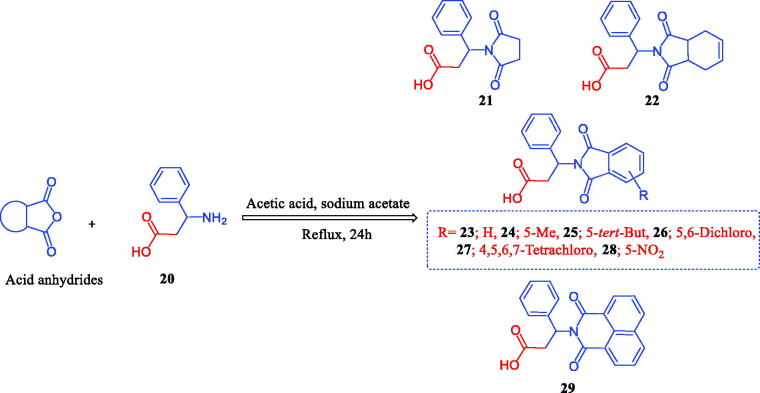
Synthesis of 1,3-isoindolinediones incorporating β-phenylalanine **21–29**.

The chemistry for the synthesis of the target molecules **2–10**, **11–19**, and **21–29** is shown in Schemes 1–3. The rationale used for the synthesis of these compounds is based on two routes where a non-carboxylic moiety, such as 3-benzenesulfonamide and hydroxyiminoethyl (oxime), and a carboxylic moiety, such as an amino acid (β-phenylalanine), are inserted into a versatile cyclic imides to explore and compare the efficacy of the substituted cyclic imides for inhibiting COX-1/COX-2 and evaluating their anti-inflammatory activity.

Non-carboxylic cyclic imide-based 3-benzenesulfonamides **2–10** and oximes **11–19** were synthesised as indicated in Schemes 1 and 2. Different carboxylic acid anhydrides were refluxed with 3-aminobenzenesulfonamide (**1**) in glacial acetic acid in the presence of anhydrous sodium acetate^6–11^ to obtain cyclic imides **2**–**10** of good yields (Scheme 1). Scheme 2 displays the process used to obtain the 2-[4-(1-(hydroxyimino)ethyl)phenyl] derivatives **11–19** via the stirring of 2-(4-acetylphenyl)-1,3-isoindolinediones and hydroxylamine hydrochloride in glacial acetic acid containing anhydrous sodium acetate^6b^. The acid imides **21–29** were synthesised as indicated in Scheme 3. The 3-(1,3-dioxoisoindolin-2-yl)-3-phenylpropanoic acid derivatives **21–29** were obtained with 66–93% yield by refluxing the cyclic imides and β-phenylalanines in glacial acetic acid containing anhydrous sodium acetate (Scheme 3). The molecular structures of the newly synthesised compounds **2–10**, **11–19**, and **21–29** were verified by spectral analyses including mass, IR, ^1^H NMR, and ^13^C NMR spectra as indicated in the experimental section.

### 2.2. Biological activity

#### 2.2.1. *In vivo* anti-inflammatory activity and SAR study

Twenty-seven compounds, **2–10**, **11–19**, and **21–29** and the reference drugs, diclofenac and celecoxib, were administered to the rat carrageenan-induced foot paw oedema model to explore the *in vivo* anti-inflammatory activities of such compounds. The percentage inhibition of paw oedema was calculated after 2 h of carrageenan treatment when maximal inhibition of carrageenan-induced paw oedema was observed (Table 1)^3^^,^^16^^,^^17^.

**Table 1.** Results of anti-inflammatory activity of the tested compounds against carrageenan induced rat paw oedema in rats^a^.

Compound No.	Mean %^a^ increase in paw weight ± SEM^b^	% Inhibition of paw oedema from control group	Compound No.	Mean %^a^ increase in paw weight ± SEM^b^	% Inhibition of paw oedema from control group
**2**	53.2 ± 0.4	71.2 ± 0.5	**17**	135.6 ± 0.8	25.1 ± 0.6
**3**	42.4 ± 0.2	77.0 ± 0.6	**18**	31.6 ± 0.4	82.9 ± 0.8
**4**	41.0 ± 0.2	77.8 ± 0.6	**19**	57.6 ± 0.5	68.8 ± 0.4
**5**	78.1 ± 0.7	57.7 ± 0.5	**21**	98.9 ± 0.6	46.4 ± 0.3
**6**	106.1 ± 0.9	40.9 ± 0.6	**22**	69.1 ± 0.5	62.9 ± 0.4
**7**	129.7 ± 0.9	29.7 ± 0.2	**23**	65.6 ± 0.3	64.4 ± 0.7
**8**	134.0 ± 0.9	26.9 ± 0.5	**24**	123.4 ± 0.9	33.1 ± 0.4
**9**	35.6 ± 0.1	80.9 ± 0.9	**25**	129.0 ± 1.1	29.6 ± 0.7
**10**	93.7 ± 0.4	49.2 ± 0.6	**26**	136.3 ± 0.8	24.4 ± 0.3
**11**	45.0 ± 0.3	75.6 ± 0.6	**27**	141.6 ± 1.0	21.2 ± 0.9
**12**	37.5 ± 0.5	79.7 ± 0.4	**28**	100.9 ± 0.8	42.5 ± 0.2
**13**	36.2 ± 0.2	80.4 ± 0.7	**29**	130.8 ± 0.7	27.0 ± 0.1
**14**	70.8 ± 0.6	61.6 ± 0.5	Celecoxib	27.3 ± 0.4	85.6 ± 0.4
**15**	95.7 ± 0.9	48.1 ± 0.2	diclofenac	30.1 ± 0.3	83.4 ± 0.5
**16**	115.3 ± 1.2	39.6 ± 0.4			

^a^Result of control group (%): 165.6 ± 1.6. ^b^Significant difference from control and celecoxib-treated group using unpaired Student’s *t*-test *p* < 0.05.

Data in Table 1 show that most of the designed compounds decreased paw oedema by 21.2–82.9% (Table 1). The reference drugs, diclofenac and celecoxib, caused 83.4 and 85.6% oedema inhibition, respectively. The non-carboxylic derivatives **2–10** (sulphonamide) and **11–19** (oxime) were generally more potent than the corresponding carboxylic acid derivatives **21–29** (β-phenylalanine), as observed for the non-carboxylic cyclic imides **2–4**, **9**, **11–13**, and **18**. These derivatives displayed the highest anti-inflammatory activities with 71.2–82.9% paw oedema reduction compared to the acidic derivatives **21–23** and **28**, with 42.5–64.4% paw oedema reduction. For sulphonamide derivatives **2–10**, the succinimide derivative **2** displayed a greater anti-inflammatory activity than the naphthalimide derivative **10**, with percentage paw oedema reduction of 71.2 and 49.2%, respectively. The conversion of succinimide **2** to the corresponding tetrahydrophthalimide **3** or phthalimide **4** led to an increase in the anti-inflammatory activity, with percentage paw oedema reduction of 77.0 and 77.8%, respectively. Electronic substitution on the phthalimide core has a direct effect on the anti-inflammatory effects. For example, the presence of the methyl or *tert*-butyl group at the 5-position led to a decrease in activity, as observed for compounds **5** and **6**, which had a percentage paw oedema reduction of 57.7 and 40.9%, respectively. Replacing the alkyl group with halogen atoms resulted in derivatives with weak anti-inflammatory activity as observed for compounds **7** and **8** (29.7 and 26.9% paw oedema reduction, respectively). However, modify 5-methyl group with 5-nitro derivative resulted in a sharp increase in the anti-inflammatory effects, as observed for compounds **5** and **9**, with 57.7 and 80.9% paw oedema reduction, respectively. Interestingly, converting the 3-benzenesulfonamides **2–10** (26.9–80.9% oedema inhibition) into the corresponding oxime derivatives **11–19** maintained the levels of the anti-inflammatory activity (25.1–82.9% oedema inhibition). In the latter compounds **11–19**, it was evident that the derivatives containing succinimide **11**, tetrahydrophthalimide **12**, phthalimide **13**, and 5-nitro phthalimide **18** had anti-inflammatory activity levels (75.6, 79.7, 80.4, and 82.9% oedema inhibition, respectively) higher than the corresponding analogues **14–17** and **19** (61.6, 48.1, 39.6, 25.1, and 68.8% oedema inhibition, respectively). Replacing 3-benzenesulfonamide with compounds that had a terminal carboxylic moiety at the cyclic imide core **21–29** caused a decrease in anti-inflammatory levels, with percentage oedema inhibition ranging from 21.2–64.4%. Similarly, the phthalimide derivative **23** had a higher activity than succinimide **21** or naphthalimide **29**, with percentage paw oedema reduction of 64.4, 46.4, and 27.0%, respectively. In contrast to derivatives **3**, **9**, **13**, and **18**, introducing a nitro group, as observed for compound **23**, on the phthalimide core resulted in derivative **28**, with a 42.5% decrease in oedema inhibition. Finally, cyclic imides with 3-benzenesulfonamide **2–10** or oxime **11–19** fragments had the greatest levels of anti-inflammatory activity, which were almost similar to those of diclofenac and celecoxib, indicating that both derivatives exerted similar interactions at the COX receptor binding site.

Table 2 shows the three graded doses of the anti-inflammatory activities of the most active cyclic imides **4**, **9**, **12**, **13**, **18**, and celecoxib.

**Table 2.** Results of anti-inflammatory activity of compounds **4**, **9**, **12**, **13**, **18**, and celecoxib against carrageenan induced rat paw oedema in rats at three graded doses.

Compound no.	Dose (mg/kg)	Percentage inhibition of paw oedema from control group	ED_50_ (mg/kg)
**4**	45	65.0	45.3
	65	77.8	
	80	86.2	
**9**	45	69.8	37.0
	65	80.9	
	80	90.5	
**12**	45	65.3	39.2
	65	79.7	
	80	87.4	
**13**	45	67.9	37.5
	65	80.4	
	80	91.1	
**18**	45	70.1	35.4
	65	82.9	
	80	91.9	
Celecoxib	45	69.8	34.1
	65	85.6	
	80	93.5	

#### 2.2.2. Ulcerogenicity

The levels of ulcerogenic activity for the most active cyclic imides **4**, **9**, **12**, **13**, **18**, along with the reference drugs, diclofenac and celecoxib, were determined according to the reported technique (Table 3)^6^^a,^^17^^,^^18^. Evidently, the phthalimide derivatives **9** and **18** had very low levels of ulcerogenic activity relative to diclofenac and celecoxib, whereas phthalimides **4**, **12**, and **13** had an ulcerogenic level similar to celecoxib and less than that of diclofenac (Table 3).

**Table 3.** Ulcerogenic potential of the tested compounds **4**, **9**, **12**, **13**, **18**, diclofenac and celecoxib in mice*.

Compound no.	Average number of ulcers	Mean sum of lengths of elongated ulcer (mm ± SEM)
Control	0.0	0.0
Celecoxib	5	5.8 ± 0.77
Diclofenac	10	7.6 ± 0.89
**4**	6	4.7 ± 0.54
**9**	4	1.7 ± 0.21
**12**	5	2.4 ± 0.13
**13**	5	2.1 ± 0.23
**18**	2	0.9 ± 0.08

*Significantly less than the celecoxib and diclofenac sodium treated group using unpaired. Student’s *t*-test *p* < 0.05.

#### 2.2.3. Cytotoxicity

Six compounds were selected by the National Cancer Institute (Bethesda, MD) based on structural variations to evaluate the *in vitro* cytotoxicity of compounds **2–6** and **8**. Thereafter, the results were compared to those of the reference drug, imatinib, as shown in Table 4. These compounds, which are derivatives of cyclic imides bearing 3-benzenesulfonamides **2–6** and **8**, were administered in single doses of 10 µM in a full NCI 59 cell line panel assay. These cell lines were obtained from nine different organs including leukaemia, non-small cell lung, colon, CNS, melanoma, ovarian, renal, prostate, and breast^19^. The results are displayed in Table 4 and expressed as the percentage growth inhibition (GI %) caused by the test compounds. Based on the results, the positive cytotoxic effects (PCE) of the tested cyclic imides **2–6** and **8** at 10 μM were 2/59–5/59, while the reference drug, imatinib, had a PCE of 20/59 (Table 4).

**Table 4.** Antitumor activity of trimellitimides derivatives **2–6** and **8** presented as growth inhibition percentages (GI %) over 59 subpanel tumour cell lines.

Compound no.	59 cancer cell lines assay in one dose 10.0 μM concentration: GI%	
	PCE*	Most sensitive cell lines*
**2**	3/59	*NSC Lung Cancer* (NCI-H522: 12%), *Renal Cancer* (CAKI-1: 12%, UO-31: 13%).
**3**	3/59	*NSC Lung Cancer* (NCI-H522: 12%), *CNS Cancer* (SNB-75: 12%), *Renal Cancer* (UO-31: 16%).
**4**	3/59	*Leukaemia* (HL-60(TB): 18%), *NSC Lung Cancer* (NCI-H522: 16.0%), *Renal Cancer* (TK-10: 11%).
**5**	5/59	*NSC Lung Cancer* (HOP-92: 11%, NCI-H322M: 11%, NCI-H522: 11%), *Renal Cancer* (UO-31: 17%), *Prostate Cancer* (PC-3: 11%).
**6**	2/59	*NSC Lung Cancer* (HOP-92: 19%), *CNS Cancer* (SNB-75: 12%).
**8**	3/59	*NSC Lung Cancer* (HOP-92: 11%,), *CNS Cancer* (SNB-75: 13%), *Renal Cancer* (UO-31: 17%).
Imatinib	20/59	*Leukaemia* (MOLT-4: 18.0, PRMI-8226: 12.6, SR: 14.6), *NSC Lung Cancer* (EKVX: 15.7, NCI-H226: 10.6, NCI-H23: 17.1), *Colon Cancer* (HCT-116: 18.6, HCT-15: 11.5, HT29: 47.1), *CNS Cancer* (SF-295: 15.1, SF-539: 24.5, U251: 10.6), *Melanoma* (LOX IMVI: 11.6, SK-MEL-5: 22.3), *Renal Cancer* (A498: 13.7), *Prostate Cancer* (PC-3: 10.6, DU-145: 14.4), *Breast Cancer* (MDA-MB-231/ATCC: 11.2, T-47D: 18.6, MDA-MB-468: 29.1).

*PCE: positive cytotoxic effect which is ratio between number of cell lines with percentage growth inhibition >10% and total number of cell lines.

#### 2.2.4. COX-1/2 inhibition and SAR study

The derivatives of cyclic imides **2–6**, **9–15**, **18**, **19**, **21–24**, and **28**, which showed anti-inflammatory activity higher than the 30% oedema inhibition, were subjected to COX-1 and COX-2 inhibition assay, with the reference drug, celecoxib, using an ovine COX-1/COX-2 assay kit (Cayman Chemicals Inc., Ann Arbour, MI). The IC_50_ (μM) and the selectivity indices (SI; IC_50_ (COX-1)/IC_50_ (COX-2)) are listed in Table 5^3,10,11,20^. Data in Table 5 show the value >387.6 as a selectivity index (SI; IC_50_ (COX-1)/IC_50_ (COX-2)) of the reference drug, celecoxib, with IC_50_ values >50/0.129 μM for COX-1/COX-2. The COX-1/COX-2 assay indicated that cyclic imides bearing a 3-benzenesulfonamide or 2-[4-(1-(hydroxyimino)ethyl)phenyl] fragment, as observed for derivatives **2–5**, **9**, **11–14**, **18**, and **19**, were considered to be potent COX-2 inhibitors with IC_50_ ≅ 0.15–0.90 μM and SI ≅ >333.3 to >55.6. The results of cyclic imides containing the non-carboxylic tails **2–4**, **9**, **11–13**, **18**, and **19**, were comparable to that of the reference drug, celecoxib (IC_50_ = 0.129 μM; COX-2 (SI) > 387.6). In contrast, cyclic imides containing a carboxylic tail, as observed for derivatives **21–24** and **28**, were non-selective COX-1/2 inhibitors with IC_50_ (COX-1) ≅ 10.9 − 24.8 μM and IC_50_ (COX-2) ≅ 22.3 − 36.3 μM and SI ≅ 0.46 − 0.68.

**Table 5.** *In vitro* cyclooxygenase (COX-1/COX-2) enzyme inhibition assay and calculated selectivity indices.

Compound no.	IC_50_ (µM)^a^		
	COX-1	COX-2	SI^b^
**2**	>50	0.26	>192.3
**3**	>50	0.20	>250.0
**4**	>50	0.18	>277.8
**5**	>50	0.90	>55.6
**6**	>50	4.00	>12.5
**9**	>50	0.15	>333.3
**10**	>50	3.50	>14.3
**11**	>50	0.22	>227.3
**12**	>50	0.16	>312.5
**13**	>50	0.16	>312.5
**14**	>50	0.80	>62.5
**15**	>50	3.50	>14.3
**18**	>50	0.15	>333.3
**19**	>50	0.28	>178.6
**21**	16.60	30.30	0.55
**22**	11.60	25.10	0.46
**23**	10.90	22.30	0.49
**24**	24.80	36.30	0.68
**28**	18.20	30.00	0.61
Celecoxib	>50	0.129	>387.6

^a^IC_50_ value is the compound concentration required to produce 50% inhibition of COX-1or COX-2 for means of two determinations using an ovine COX-1/COX-2 assay kit (catalog no. 560101, Cayman Chemicals Inc., Ann Arbour, MI) and deviation from the mean is <10% of the mean value. ^b^Selectivity index (COX-1 IC_50_/COX-2 IC_50_).

The inhibitors, cyclic imides **2–4**, **9**, **11–13**, **18**, and **19**, were the most potent and active derivatives with IC_50_ values of 0.26 μM (SI > 192.3), 0.20 μM (SI > 250.0), 0.18 μM (SI > 277.8), 0.15 μM (SI > 333.3), 0.22 μM (SI > 227.3), 0.16 μM (SI > 312.5), 0.16 μM (SI > 312.5), 0.15 μM (SI > 333.3), and 0.28 μM (SI > 178.6), respectively, relative to celecoxib which had an IC_50_ value of 0.129 μM and SI value of >387.6. The cyclic imides incorporating oximes, as observed for compounds **11–14** and **19** (COX-2 (SI) >62.5 − 312.5), were more active than the corresponding cyclic imides bearing a 3-benzenesulfonamide tail, as observed for compounds **2–5** and **10** (COX-2 (SI) ≫14.3 − 277.8). Interestingly, compounds **9** and **18** containing the 3-benzenesulfonamide and oxime tails, respectively, had the same COX-2 (SI) value >333.3, which may be attributed to the same binding interaction with the COX-2 putative pocket. The presence of the carboxylic moieties on the cyclic imides, as observed for series **21–24** and **28**, afforded non-selective COX-1/COX-2 inhibition with a relatively high COX-1 inhibition of IC_50_ ≅ 10.9–24.8 μM) and lower COX-2 inhibition of IC_50_ ≅ 22.3–36.3 μM). Compounds **22** and **23** had low COX-2 inhibition, with IC_50_ values of 25.1 and 22.3 μM, respectively, and high COX-1 inhibition, with IC_50_ values of 11.6 and 10.9 μM, respectively, compared to their 3-benzenesulfonamide and oxime analogues **3**, **4**, **12**, and **13**, with SI values of >250.0, 277.8, 312.5, and >312.5, respectively. Hence, the sulphonamide (–SO_2_NH_2_) and oxime (–C=NOH) fragments are important pharmacophores for the interaction with the COX-2 binding pocket.

The structure-activity relationships (SAR) of the test compounds **2–6**, **9–15**, **18**, **19**, **21–24**, and **28** in the COX-1/2 inhibition assay revealed the following: (i) generally, the COX-2 isozyme was potentially affected by cyclic imides **2–5**, **9**, **11–14**, **18**, and **19**, with IC_50_ values ranging from 0.15 − 0.90 μM and SI ≅ >333.3 to >55.6 compared to celecoxib (IC_50_ = 0.129 μM; COX-2 (SI) > 387.6); (ii) the non-carboxylic cyclic imides, including the 3-benzenesulfonamide and oxime tails **2–5**, **9**, **11–14**, **18**, and **19**, were potent inhibitors of the COX-2 isozyme, while the carboxylic cyclic imide derivatives **21–24** and **28** were very weak or non-COX-2 inhibitors. Consequently, the sulphonamide (SO_2_NH_2_) and oxime (–C=NOH) fragments are an essential part of COX-2 inhibition and recognition; (iii) the cyclic imides with a nitro (NO_2_) group at the 5-position in compounds containing sulphonamide and oxime fragments, as observed for compounds **9** and **18**, possessed the strongest COX-2 inhibitory activity among the tested compounds (IC_50_ = 0.15 μM and SI > 333.3); (iv) in contrast, the carboxylic cyclic imide substituted with a nitro (NO_2_) group at the 5-position, as observed for compound **28**, had reduced COX-2 inhibitory activity (IC_50_ = 30.0 μM) and increased COX-1 inhibitory activity (IC_50_ = 18.2 μM and SI = 0.61); (v) the tetrahydrophthalimide and phthalimide core structures, as observed for compounds **3** (IC_50_ = 0.20 μM and SI > 250), **4** (IC_50_ = 0.18 μM and SI > 277.8), **12** (IC_50_ = 0.16 μM and SI > 312.5), and **13** (IC_50_ = 0.16 μM and SI > 312.5), had higher inhibitory activity than the succinimide derivatives, as observed for compounds **2** (IC_50_ = 0.26 μM and SI > 192.3) and **11** (IC_50_ = 0.22 μM and SI > 227.3). These aforementioned results indicate the importance of the double bonds for COX-2 inhibition; (vi) replacing the phthalimide fragments in compounds **4** and **13** (IC_50_ = 0.18 μM, and 0.16 μM, respectively), with the naphthalimide structure in compounds **10** and **19** decreasing the COX-2 inhibitory activity with IC_50_ of 3.5 μM and 0.28 μM, respectively; (vii) substituting the phthalimide core in compounds **4** (IC_50_ = 0.18 μM), **13** (IC_50_ = 0.16 μM) and **23** (IC_50_ = 19.3 μM) with the methyl moiety (CH_3_) at the 5-position in compounds **5**, **14**, and **24** resulted in a decrease in COX-2 inhibition (IC_50_ = 0.9 μM, 0.8 μM, and 36.30 μM, respectively) and an increase in COX-1 inhibition of compound **24** (IC_50_ = 24.8 μM); (viii) replacing the methyl fragment (CH_3_) in compounds **5** and **14** (IC_50_ = 0.9 μM, and 0.8 μM, respectively) with the bulky *tert*-butyl group (–C(CH_3_)_3_) in compounds **6** and **15** led to a decrease in COX-2 inhibition (IC_50_ = 4.0 μM, and 3.5 μM, respectively); (ix) converting the terminal sulphonamide or oxime fragments in compounds **3** (IC_50_ = 0.2 μM), **4** (IC_50_ = 0.18 μM), **12** (IC_50_ = 0.16 μM), and **13** (IC_50_ = 0.16 μM) into a carboxylic acid (–COOH) moiety containing the same cyclic imide scaffold found in compounds **22** and **23** resulted in a decrease in COX-2 inhibition (IC_50_ = 25.1 μM and 22.3 μM, respectively) and increase in COX-1 inhibition (IC_50_ = 11.6 μM and 10.9 μM, respectively). Briefly, the non-carboxylic cyclic imide scaffolds containing the 3-benzenesulfonamide or 2-[4-(1-(hydroxyimino)ethyl)phenyl] fragments, as observed for derivatives **2–5**, **9**, **11–14**, **18**, and **19**, were the most active inhibitors of the COX-2 isozyme and some displayed higher levels of selective COX-2 inhibition (SI ≥ 250 − 333.3), which were comparable to levels for celecoxib (SI ≥ 387.6).

### 2.3. Molecular docking studies

The molecular modelling technique was used to establish and understand the binding mode of the most bioactive compounds^21^^,^^22^. The selectivity of the cyclic imide analogues towards COX-2 was studied using a molecular docking protocol on the MOE 2008.10 programme obtained from Chemical Computing Group Inc. (Montreal, Canada)^23^.

**Figure 2.**
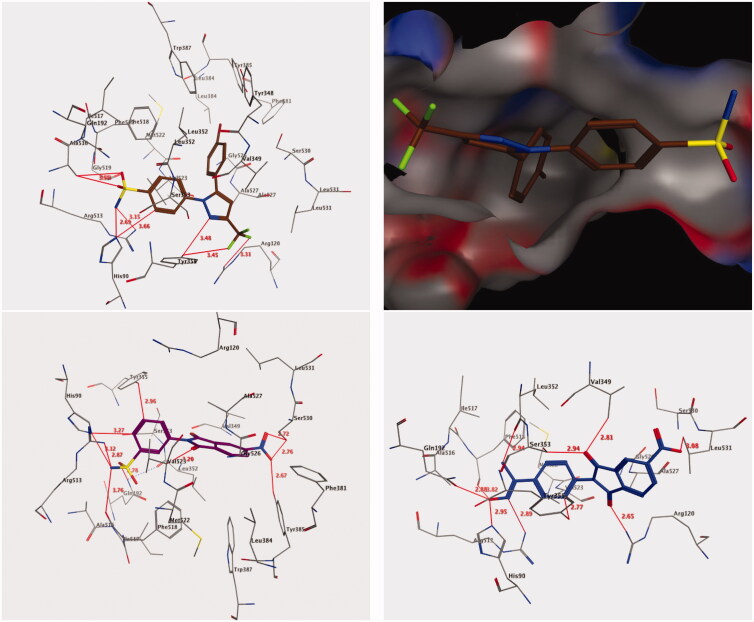
3D interactions of compounds SC-558 ligand (upper panel), **9** (lower left panel), and **18** (lower right panel) with the active site of COX-2. Hydrogen bonds are depicted as red lines.

Molecular docking was performed to examine the best interaction between the most active compounds, **9** and **18**, and the COX-2 pocket binding site (Figure 2, lower panels). The crystallographic binding site on the COX-2 isozyme in complex with the SC-558 ligand, an analogue of celecoxib, was derived from Protein Data Bank (PDB code: 1CX2) (Figure 2, upper panels)^4^.

The scoring function and the hydrogen bond formed among these compounds and the surrounding amino acids were used to predict the interaction mode of compounds **9** and **18** in the putative active pocket of the COX-2 isozyme. Figure 2 shows the results of the docking studies for both compounds **9** and **18**, which possess the common cyclic imide pharmacophore and a similar binding interaction, including H-bonding and hydrophobic interactions within the putative binding pocket. These compounds were inserted deep into the hydrophilic site of the COX-2 isozyme where the sulphonamide and oxime groups can interact with the hydrophilic pocket (His90, Gln192, and Arg513) through a network of hydrogen bonds; such binding interactions were similar to that of SC-558 co-crystallized in the COX-2 active site^4^. Additionally, the cyclic imide cores of compounds **9** and **18** were oriented at the top of the channel, which were involved in the hydrophobic interaction with amino acids, Ala527, Val349, Trp387, Val523, and Leu352. The terminal sulphonamide group (–SO_2_NH_2_) was responsible for the stability of the docked compound **9** through its conserved role in the binding pocket through the formation of suitable H-bonds with the key amino acids, Arg513 (2.87 Å), His90 (3.12 Å), Gln192 (2.78 Å), and Phe518 (3.76 Å) [Figure 2, lower left panel]. Meanwhile, the phenyl ring of the 3-benzenesulfonamide formed two non-classical H-bonds by binding with amino acids, His90 (3.27 Å) and Tyr355 (2.96 Å), and an additional hydrophobic interaction with Ser353 (3.95 Å) through CH-pi interactions. The 5-nitro group of cyclic imide was also identified to form three H-bonds with the amino acids Tyr385 (2.67 Å) and Ser530 (2.76 Å and 2.72 Å), whereas one of the carbonyl group of cyclic imide interacted with the amino acid Val523 through a non-classical H-bond (3.20 Å) [Figure 2, lower left panel].

In a similar manner, the complex generated by the docking of compound **18** [Figure 2, lower right panel] showed that the terminal oxime moiety (C=N–OH) formed H-bonds with amino acids Arg513 (2.89 Å), His90 (2.95 Å), Gln192 (2.88 Å), and Phe518 (3.82 Å) similar to the sulphonamide moiety in compound **9** [Figure 2, lower right panel]. Additionally, the methyl moiety of the oxime structure interacted with amino acid Leu352 through a non-classical H-bond (2.94 Å), while the N-phenyl moiety formed another non-classical H-bond with Tyr355 (2.77 Å) [Figure 2, lower right panel]. Moreover, the two carbonyl groups of cyclic imide were held by one classical and two non-classical H-bonds with amino acids Arg120 (2.65 Å), Ser353 (2.94 Å), and Val349 (2.81 Å) [Figure 2, lower right panel]. Finally, the 5-nitro group of cyclic imide formed a non-classical H-bond with Leu531 (3.08 Å). The binding interactions described herein are typical of the pyrazolic prototype (SC-558), which is a selective inhibitor of COX-2. Altogether, such findings confirm the molecular design of the reported class of anti-inflammatory 1,3-isoindoledione scaffolds^10^^,^^11^.

## 3. Conclusions

The cyclic imide scaffolds bearing the non-carboxylic 3-benzenesulfonamide or 2-[4-(1-(hydroxyimino)ethyl)phenyl] fragments **2**–**10**, and **11–19** and the carboxylic derivatives **21–29** were synthesised and evaluated for their *in vivo* anti-inflammatory and ulcerogenic activities and *in vitro* cytotoxicity. The derivatives revealed as the most active anti-inflammatory agents were also subjected to an inhibition assay with the COX-1/COX-2 enzymes. Among the tested compounds, cyclic imide derivatives bearing 3-benzenesulfonamides (**2–4**, **9**), acetophenone oximes (**11**, **12**, **13**), and β-phenylalanine (**18**) exhibited remarkable anti-inflammatory activities (71.2–82.9% oedema inhibition) compared to the reference drugs, celecoxib and diclofenac (85.6 and 83.4% oedema inhibition, respectively). Cyclic imides attached to 3-benzenesulfonamide or oxime were stronger anti-inflammatory agents and selective COX-2 inhibitors than the carboxylic acid derivatives containing the same scaffolds. Compounds **2–4**, **9**, **11–13**, **18**, and **19** were the most potent COX-2 inhibitors (IC_50_ = 0.26, 0.20, 0.18, 0.15, 0.22, 0.16, 0.16, 0.15, and 0.28 μM, respectively) and demonstrated values comparable to that of celecoxib (IC_50_ = 0.129 μM). Some cyclic imide derivatives **2–6** and **8** were subjected to cytotoxic evaluation which showed weak positive cytotoxic effects (PCE = 2/59–5/59) compared to the standard drug, imatinib (PCE = 20/59). From the COX-2 inhibition assay and docking results, compounds **9** and **18** were recognised as the most active analogues, with the highest recognition at the COX-2 binding site and a correlation identified with the COX-2 selectivity indices.

## 4. Experimental

### 4.1. Chemistry

Melting points (uncorrected) were recorded on a Barnstead 9100 Electrothermal melting apparatus (APS Water Services Corporation, Van Nuys, CA) while the IR spectra were recorded on a FT-IR Perkin-Elmer spectrometer (PerkinElmer Inc., Waltham, MA). The ^1^H NMR and ^13^C NMR were measured in DMSO-d_6_ or CDCl_3_, on Bruker 700 or 500 and 176 or 125 MHz instruments, respectively (Bruker, Billerica, MA). Chemical shifts are reported in *δ* ppm. Mass spectra were recorded on an Agilent 6320 Ion Trap mass spectrometer (Agilent Technologies, Santa Clara, CA). C, H, and N were analysed at the Research Centre, College of Pharmacy, King Saud University, Saudi Arabia. The results were within ±0.4% of the theoretical values. Compounds **4**, **8**, **9**, **12–17**, and **22–24** were prepared according to a previously reported procedure^6^^b,^^24^.

#### 4.1.1. General procedure for the synthesis of 1,3-isoindolediones 2–10

A mixture of an equimolar amount of 3-benzenesulfonamide and acid anhydrides (5.0 mmol) was heated under reflux for 12 h in glacial acetic acid (15 ml) containing anhydrous sodium acetate (0.69 g, 5.0 mmol) (Scheme 1). The reaction mixture was cooled, filtered, and the obtained solid was washed with water, dried, and re-crystallized.

##### 4.1.1.1. 3-(2,5-Dioxopyrrolidin-1-yl)benzenesulfonamide (2)

M.P. 195–197°C, 90% yield (Ethanol); IR (KBr, cm^−1^) *ν*: 3337, 3246 (NH_2_), 1696 (C=O), 1339, 1157 (O = S=O); ^1^H NMR (500 MHz, DMSO-d_6_): *δ* 2.82 (s, 4H), 7.37 (s, 2H), 7.45 − 7.47 (t, 1H, *J* = 7.9 Hz), 7.60 − 7.63 (t, 1H, *J* = 7.9 Hz), 7.82 (s, 1H), 7.88 − 7.90 (d, 1H, *J* = 8.0 Hz); ^13^C NMR (125 MHz, DMSO-d_6_): *δ* 28.82, 124.78, 125.87, 129.69, 130.46, 133.19, 145.19, 176.68; C_10_H_10_N_2_O_4_S: *m*/*z* (254.3).

##### 4.1.1.2. 3-(1,3-Dioxo-1,3,3a,4,7,7a-hexahydro-2H-isoindol-2-yl)benzenesulfonamide (3)

M.P. 159–161°C, 91% yield (Ethanol); IR (KBr, cm^−1^) *ν*: 3293, 3125 (NH_2_), 1773, 1699 (C=O), 1341, 1160 (O=S=O); ^1^H NMR (700 MHz, DMSO-d_6_): *δ* 2.30–2.32 (d, 2H, *J* = 12.6 Hz), 2.48–2.51 (d, 2H, *J* = 16.1 Hz), 3.35 (s, 2H), 5.98 (s, 2H), 7.46–7.47 (d, 1H, *J* = 7.0 Hz), 7.52 (s, 2H), 7.70 (s, 2H), 7.88–7.89 (d, 1H, *J* = 6.3 Hz); ^13^C NMR (176 MHz, DMSO-d_6_): *δ* 23.71, 40.35, 124.52, 126.03, 128.23, 130.26, 130.73, 133.22, 145.35, 179.55; C_14_H_14_N_2_O_4_S: *m*/*z* (306.3).

##### 4.1.1.3. 3-(5-Methyl-1,3-dioxoisoindolin-2-yl)benzenesulfonamide (5)

M.P. 225–226°C, 86% yield (Methanol); IR (KBr, cm^−1^) *ν*: 3308, 3228 (NH_2_), 1779, 1717 (C=O), 1339, 1164 (O=S=O); ^1^H NMR (700 MHz, DMSO-d_6_): *δ* 2.54 (s, 3H), 7.55 (s, 2H), 7.69–7.71 (d, 1H, *J* = 7.8 Hz), 7.73–7.76 (q, 2H, *J* = 7.9 Hz), 7.82 (s, 1H), 8.87–7.91 (q, 2H, *J* = 7.8 Hz), 7.95 (s, 1H); ^13^C NMR (176 MHz, DMSO-d_6_): *δ* 21.89, 123.95, 124.40, 124.76, 125.57, 129.34, 130.12, 130.98, 132.31, 132.82, 135.69, 145.29, 146.35, 167.19, 167.29; C_15_H_12_N_2_O_4_S: *m*/*z* (316.4).

##### 4.1.1.4. 3-(5-(tert-Butyl)-1,3-dioxoisoindolin-2-yl)benzenesulfonamide (6)

M.P. 151–153°C, 79% yield (Methanol); IR (KBr, cm^−1^) *ν*: 3354, 3264 (NH_2_), 1777, 1718 (C=O), 1375, 1164 (O=S=O); ^1^H NMR (700 MHz, DMSO-d_6_): *δ* 1.39 (s, 9H), 7.57 (s, 2H), 7.69–7.70 (d, 1H, *J* = 7.8 Hz), 7.74–7.77 (t, 1H, *J* = 7.8 Hz), 7.91–7.95 (q, 3H, *J* = 7.8 Hz), 7.96–7.97 (d, 2H, *J* = 6.4 Hz); ^13^C NMR (125 MHz, DMSO-d_6_): *δ* 31.26, 36.03, 120.81, 123.98, 124.83, 125.64, 129.46, 130.17, 131.09, 132.19, 132.27, 132.84, 145.28, 159.10, 167.09, 167.42; C_18_H_18_N_2_O_4_S: *m*/*z* (358.3).

##### 4.1.1.5. 3-(5,6-Dichloro-1,3-dioxoisoindolin-2-yl)benzenesulfonamide (7)

M.P. 256–258°C, 71% yield (Methanol); IR (KBr, cm^−1^) *ν*: 3338, 3240 (NH_2_), 1779, 1718 (C=O), 1330, 1154 (O = S=O); ^1^H NMR (500 MHz, DMSO-d_6_): *δ* 7.50 (s, 2H), 7.65–7.66 (d, 1H, *J* = 8.3 Hz), 7.70–7.73 (t, 1H, *J* = 7.9 Hz), 7.92–7.93 (d, 1H, *J* = 7.9 Hz), 7.95–7.96 (t, 1H, *J* = 3.5 Hz), 8.21 (s, 2H); ^13^C NMR (125 MHz, DMSO-d_6_): *δ* 124.69, 125.93, 125.98, 130.01, 130.58, 131.76, 132.27, 138.37, 145.44, 165.27; C_14_H_8_Cl_2_N_2_O_4_S: *m*/*z* (371.2).

##### 4.1.1.6. 3-(1,3-Dioxo-1H-benzo[de]isoquinolin-2(3H)-yl)benzenesulfonamide (10)

M.P. 241–243°C, 76% yield (Methanol); IR (KBr, cm^−1^) *ν*: 3367, 3221 (NH_2_), 1776, 1711 (C=O), 1336, 1148 (O=S=O); ^1^H NMR (500 MHz, DMSO-d_6_): *δ* 7.39 (s, 2H), 7.52–7.53 (d, 1H, *J* = 7.8 Hz), 7.82–7.85 (m, 2H), 8.38–8.40 (d, 2H, *J* = 8.3 Hz), 8.43–7.45 (d, 2H, *J* = 8.3 Hz), 8.50–8.54 (m, 3H); ^13^C NMR (125 MHz, DMSO-d_6_): *δ* 118.94, 122.73, 125.96, 126.99, 127.32, 127.73, 129.76, 131.33, 133.15, 134.92, 135.85, 160.77, 163.97; C_18_H_12_N_2_O_4_S: *m*/*z* (352.3).

#### 4.1.2. General procedure for the synthesis of 1,3-isoindoledione 11–19

A mixture of 1,3-isoindolinediones (5.0 mmol) and hydroxylamine hydrochloride (0.56 g, 8 mmol) was stirred at room temperature for 24 h in glacial acetic acid (20 ml) (Scheme 2). The reaction mixture was then filtered and the obtained solid was washed with water, dried, and re-crystallized.

##### 4.1.2.1. 1-(4-(1-(Hydroxyimino)ethyl)phenyl)pyrrolidine-2,5-dione (11)

M.P. 184–186°C, 89% yield (Methanol); IR (KBr, cm^−1^) *ν*: 3358 (OH), 1774, 1690 (C=O), 1634 (C=N); ^1^H NMR (700 MHz, DMSO-d_6_): *δ* 2.12 (s, 3H), 2.52–2.54 (t, 2H, *J* = 6.7 Hz), 2.57–2.59 (t, 2H, *J* = 6.4 Hz), 7.58–7.61 (q, 4H, *J* = 9.5 Hz), 10.08 (s, 1H); ^13^C NMR (176 MHz, DMSO-d_6_): *δ* 11.83, 29.20, 31.50, 119.03, 131.94, 140.10, 152.95, 170.70, 174.32; C_12_H_12_N_2_O_3_: *m*/*z* (232.1).

##### 4.1.2.2. 2-(4-(1-(Hydroxyimino)ethyl)phenyl)-5-nitroisoindoline-1,3-dione (18)

M.P. 269–271°C, 92% yield (Ethanol); IR (KBr, cm^−1^) *ν*: 3428 (OH), 1737, 1699 (C=O), 1645 (C=N); ^1^H NMR (700 MHz, DMSO-d_6_): *δ* 2.21 (s, 3H), 7.47–7.48 (d, 2H, *J* = 8.4 Hz), 7.81–7.82 (d, 2H, *J* = 8.3 Hz), 8.12–8.14 (t, 1H, *J* = 7.8 Hz), 8.26–8.27 (d, 1H, *J* = 7.4 Hz), 8.34–8.35 (d, 1H, *J* = 7.1 Hz), 11.39 (s, 1H); ^13^C NMR (176 MHz, DMSO-d_6_): *δ* 12.04, 123.37, 126.53, 127.55, 127.79, 128.88, 132.06, 134.01, 136.90, 137.45, 144.98, 152.95, 163.02, 165.60; C_16_H_11_N_3_O_5_: *m*/*z* (325.1).

##### 4.1.2.3. 2-(4-(1-(Hydroxyimino)ethyl)phenyl)-1H-benzo[de]isoquinoline-1,3(2H)-dione (19)

M.P. >300°C, 92% yield (Ethanol); Yield, 87%; IR (KBr, cm^−1^) *ν*: 3090 (OH), 1775, 1716 (C=O), 1649 (C=N); ^1^H NMR (500 MHz, DMSO-d_6_): *δ* 2.22 (s, 3H), 7.29–7.31 (t, 2H, *J* = 6.5 Hz), 7.76–7.77 (t, 2H, *J* = 6.5 Hz), 7.81–7.84 (t, 2H, *J* = 7.5 Hz), 8.38–8.40 (d, 2H, *J* = 8.5 Hz), 8.51–8.52 (d, 2H, *J* = 6.5 Hz), 11.14 (s, 1H); ^13^C NMR (125 MHz, DMSO-d_6_): *δ* 11.93, 122.82, 126.43, 127.29, 129.13, 129.53, 130.46, 131.27, 134.75, 136.01, 137.72, 146.22, 152.80, 162.82, 164.02; C_20_H_14_N_2_O_3_: *m*/*z* (330.1).

#### 4.1.3. General procedure for the synthesis of 1,3-isoindoledione 21–29

A mixture of an equimolar amount of β-phenylalanine (0.83 gm, 5.0 mmol) and acid anhydrides (5.0 mmol) was heated under reflux for 24 h in glacial acetic acid (20 ml) containing anhydrous sodium acetate (0.69 g, 5.0 mmol) (Scheme 3). The reaction mixture was cooled and filtered, and the obtained solid was washed with water, dried, and re-crystallized.

##### 4.1.3.1. 3-(2,5-Dioxopyrrolidin-1-yl)-3-phenylpropanoic acid (21)

M.P. 259–261°C, 93% yield (Ethanol); IR (KBr, cm^−1^) *ν*: 3111 (OH), 1743, 1702 (C=O); ^1^H NMR (700 MHz, DMSO-d_6_): *δ* 2.44–2.49 (m, 2H), 2.52–2.57 (m, 2H), 3.24–3.27 (dd, 1H, *J* = 11.9, 14.0 Hz), 3.37–3.39 (dd, 1H, *J* = 4.5, 14.1 Hz), 4.59–4.61 (dd, 1H, *J* = 4.5, 11.7 Hz), 7.09–7.10 (d, 2H, *J* = 7.3 Hz), 7.15–7.17 (t, 1H, *J* = 7.3 Hz), 7.22–7.24 (t, 2H, *J* = 7.5 Hz); ^13^C NMR (176 MHz, DMSO-d_6_): *δ* 28.02, 34.06, 55.53, 126.60, 128.68, 129.05, 139.32, 170.38, 177.55; C_13_H_13_NO_4_: *m*/*z* (247.2).

##### 4.1.3.2. 3-(5-(tert-Butyl)-1,3-dioxoisoindolin-2-yl)-3-phenylpropanoic acid (25)

M.P.(0).119–121°C, 76% yield (Methanol); IR (KBr, cm^−1^) *ν*: 3219 (OH), 1739, 1703 (C=O); ^1^H NMR (700 MHz, DMSO-d_6_): *δ* 1.32 (s, 9H), 3.43–3.47 (t, 1H, *J* = 12.8 Hz), 3.52–3.55 (dd, 1H, *J* = 4.0, 14.4 Hz), 4.57–4.60 (dd, 1H, *J* = 4.0, 12.5 Hz), 7.06–7.08 (t, 1H, *J* = 7.3 Hz), 7.10–7.11 (d, 2H, *J* = 7.3 Hz), 7.15–7.18 (t, 2H, *J* = 7.6 Hz), 7.67–7.69 (d, 1H, *J* = 7.8 Hz), 7.72 (s, 1H), 7.79–7.81 (dd, 1H, *J* = 1.5, 7.9 Hz); ^13^C NMR (176 MHz, DMSO-d_6_): *δ* 31.22, 35.35, 35.83, 57.41, 119.93, 123.04, 126.28, 128.65, 128.80, 129.60, 131.50, 132.38, 140.75, 158.31, 168.60, 168.89, 174.40; C_21_H_21_NO_4_: *m*/*z* (351.2).

##### 4.1.3.3. 3-(5,6-Dichloro-1,3-dioxoisoindolin-2-yl)-3-phenylpropanoic acid (26)

M.P. 205–207°C, 89% yield (Ethanol); IR (KBr, cm^−1^) *ν*: 2890 (OH), 1742, 1717 (C=O); ^1^H NMR (500 MHz, DMSO-d_6_): *δ* 3.28–3.33 (dd, 1H, *J* = 12.0, 14.0 Hz), 3.47–3.51 (dd, 1H, *J* = 4.7, 14.1 Hz), 5.12–5.15 (dd, 1H, *J* = 4.8, 11.7 Hz), 7.11–7.19 (m, 5H), 8.16 (s, 2H), 13.44 (s, 1H); ^13^C NMR (125 MHz, DMSO-d_6_): *δ* 34.33, 53.86, 126.19, 127.09, 128.82, 129.17, 130.81, 137.58, 138.56, 165.67, 170.17; C_17_H_11_Cl_2_NO_4_: *m*/*z* (364.2).

##### 4.1.3.4. 3-Phenyl-3-(4,5,6,7-tetrachloro-1,3-dioxoisoindolin-2-yl)propanoic acid (27)

M.P. 280–282°C, 93% yield (Methanol); IR (KBr, cm^−1^) *ν*: 2870 (OH), 1741, 1715 (C=O); ^1^H NMR (500 MHz, DMSO-d_6_): *δ* 3.28–3.33 (t, 1H, *J* = 12.0 Hz), 3.49–3.53 (dd, 1H, *J* = 4.9, 14.3 Hz), 5.15–5.18 (dd, 1H, *J* = 4.7, 11.2 Hz), 7.13–7.21 (m, 5H), 13.47 (s, 1H); ^13^C NMR (125 MHz, CDCl_3_): *δ* 34.25, 54.18, 127.09, 127.48, 128.87, 129.11, 129.15, 137.53, 139.62, 163.03, 169.90; C_17_H_9_Cl_4_NO_4_: *m*/*z* (433.1).

##### 4.1.3.5. 3-(5-Nitro-1,3-dioxoisoindolin-2-yl)-3-phenylpropanoic acid (28)

M.P.(0).191–193°C, 83% yield (Ethanol); IR (KBr, cm^−1^) *ν*: 2910 (OH), 1745, 1715 (C=O); ^1^H NMR (700 MHz, DMSO-d_6_): *δ* 3.29–3.34 (dd, 1H, *J* = 4.0, 16.0 Hz), 3.49–3.52 (dd, 1H, *J* = 6.9, 13.0 Hz), 5.15–5.18 (dd, 1H, *J* = 6.9, 16.0 Hz), 7.13–7.21 (m, 5H), 8.07–8.10 (t, 1H, *J* = 11.0 Hz), 8.16–8.18 (d, 1H, *J* = 10.2 Hz), 8.31–8.33 (d, 1H, *J* = 11.3 Hz); ^13^C NMR (176 MHz, DMSO-d_6_): *δ* 34.30, 54.03, 122.39, 127.12, 127.88, 128.63, 128.85, 129.20, 129.50, 129.66, 132.76, 137.50, 137.62, 144.88, 162.86, 165.51, 169.69, 170.07; C_17_H_12_N_2_O_6_: *m*/*z* (340.1).

##### 4.1.3.6. 3-(1,3-Dioxo-1H-benzo[de]isoquinolin-2(3H)-yl)-3-phenylpropanoic acid (29)

M.P. 151–153°C, 66% yield (Methanol); IR (KBr, cm^−1^) *ν*: 2930 (OH), 1708, 1667 (C=O); ^1^H NMR (700 MHz, DMSO-d_6_): *δ* 2.92–2.95 (t, 1H, *J* = 7.7 Hz), 4.24–4.26 (t, 1H, *J* = 7.6 Hz), 4.95–4.97 (dd, 1H, *J* = 7.6, 14.5 Hz), 7.28–7.31 (q, 3H, *J* = 7.4 Hz), 7.85–7.87 (t, 1H, *J* = 7.5 Hz), 7.90–7.93 (q, 2H, *J* = 7.6 Hz), 8.44 (s, 2H), 8.47-8.48 (s, 1H, *J* = 6.9 Hz), 8.51–8.54 (t, 2H, *J* = 7.5 Hz); ^13^C NMR (176 MHz, DMSO-d_6_): *δ* 33.96, 34.82, 54.73, 119.44, 122.42, 127.69, 128.02, 128.54, 128.95, 129.10, 129.40, 131.20, 132.95, 134.83, 135.19, 135.87, 139.21, 161.18, 163.51, 163.74; C_21_H_15_NO_4_: *m*/*z* (345.1).

### 4.2. Biological evaluation

#### 4.2.1. Anti-inflammatory screening

Anti-inflammatory assessment of the newly synthesised compounds was carried out using an *in vivo* rat carrageenan-induced foot paw oedema model, as reported previously^16^^,^^17^. Compounds **6**, **7**, **8**, **10**, **11**, **18**, diclofenac, and celecoxib were tested at three different doses and their ED_50_ was determined.

#### 4.2.2. Ulcerogenicity measurement

Ulcerogenicity was evaluated according to a previously reported method^6^^a,^^17^^,^^18^. The number and total length of the ulcers for each animal were measured and their averages were calculated and used as the ulcer indices.

#### 4.2.3. *In vitro* cyclooxygenase (COX) inhibition assay

To determine the relative ability of the test compounds and reference drugs to inhibit COX-1/COX-2 isozymes, we used the colorimetric COX (ovine) inhibitor screening assay kit (Cayman Chemicals Inc., Ann Arbour, MI), according to the manufacturer’s instructions^3^^,^^10^^,^^11^^,^^20^.

### 4.3. Docking methodology

Molecular modelling studies were performed using the 2007.09 software from Chemical Computing Group Inc. (Montreal, Canada). The docking protocol was similar to that mentioned in our previous report^19^.
